# Comparative effectiveness of laparoscopic versus open colectomy in colon cancer patients: a study protocol for emulating a target trial using cancer registry data

**DOI:** 10.1007/s00432-024-06057-x

**Published:** 2025-01-11

**Authors:** Semaw Ferede Abera, Gabriele Robers, Anika Kästner, Ulrike Stentzel, Kerstin Weitmann, Wolfgang Hoffmann

**Affiliations:** 1 Institute for Community Medicine, Section Epidemiology of Health Care and Community Health, University Medicine Greifswald, 17489 Greifswald, Germany; 2 Cancer Registry Mecklenburg-Western Pomerania, 17475 Greifswald, Germany

**Keywords:** Target trial emulation, Laparoscopic colectomy, Colon cancer, Overall survival, DAGs, Bias Analysis, Germany

## Abstract

Introduction: The objective of this study is to compare the 5 year overall survival of patients with stage I–III colon cancer treated by laparoscopic colectomy versus open colectomy. Methods: Using Mecklenburg-Western Pomerania Cancer Registry data from 2008 to 2018, we will emulate a phase III, multicenter, open-label, two-parallel-arm hypothetical target trial in adult patients with stage I–III colon cancer who received laparoscopic or open colectomy as an elective treatment. An inverse-probability weighted Royston‒Parmar parametric survival model (RPpsm) will be used to estimate the hazard ratio of laparoscopic versus open surgery after confounding factors are balanced between the two treatment arms. Further to the hazard ratio, we will also compute differences in the absolute risk (at 1, 3, and 5 years) and restricted mean survival time (up to 1, 3, and 5 years). A weighted Kaplan‒Meier curve will be used to compare five-year overall survival in both treatment arms. Various comparator and sensitivity analyses will be performed to check the robustness of the results that will be estimated by the RPpsm main model. Treatment period- and stage-specific results will also be provided. Discussion: This study aims to causally model the effect of laparoscopic versus open colectomy on 5 year overall survival using a target trial emulation approach. As the cancer registry data do not cover BMI, comorbidity, and previous abdominal surgery for non-malignant indications, the potential for residual confounding arising from these factors is a limitation of this study. This will be approached in a quantitative bias analysis using the E-method. The results will substantiate existing evidence on the comparative effectiveness of laparoscopic versus open colectomy in patients with stage I–III colon cancer and may guide clinical decisions as to whether a laparoscopic approach is as safe as an open approach in terms of improving 5-year overall survival in these patient groups.

**Supplementary Information:**

The online version contains supplementary material available at 10.1007/s00432-024-06057-x.

## Introduction

Whether laparoscopic surgery can be a clinically acceptable and alternative standard treatment for patients with colon cancer depends critically on long-term oncological outcomes (Ptok et al. 2014). The effects of laparoscopic versus open surgery on overall survival (OS) and other oncologic outcomes have been evaluated in a number of randomized controlled trials (RCTs) and meta-analyses of RCTs (Bagshaw et al. 2012; Bonjer et al. 2007; Nelson et al. 2004; Buunnen et al. 2009; Deijen et al. 2017; Fleshman et al. 2007; Jayne et al. 2010; Kitano et al. 2017; Lacy et al. 2008; Lacy et al. 2002; Theophilus et al. 2014; Di et al. 2013). Some of these RCTs evaluated only short-term (3-year) OS and revealed that laparoscopic surgery is non-inferior to open surgery (Study et al. 2004; Study et al. 2009; Lacy et al. 2002). Other RCTs evaluated long-term OS and mostly demonstrated an equivalent effect [Bagshaw et al. 2012; Deijen et al. 2017; Fleshman et al. 2007; Jayne et al. 2010; Kitano et al. 2017; Lacy et al. 2008]. However, some of these studies focused only on stage II and III patients, had an age restriction, were based on a small number of patients, had the problem of incomparability of results due to the use of an unusual study design, or did not separate colon cancer from rectal cancer (Fleshman et al. 2007; Jayne et al. 2010; Kitano et al. 2017).

The available evidence from previous RCTs and meta-analyses, however, revealed inconsistent and even contradictory stage-specific outcomes. One major limitation of the above-cited RCTs was that the estimated overall and stage-specific survival comparisons were based on only small numbers of at-risk patients. Lancy et al., for instance, showed the superiority of laparoscopic surgery over open surgery in terms of OS only in patients with stage III tumors (Lacy et al. 2008; Lacy et al. 2002). This even inspired the authors to propose a new hypothesis on how the less invasive surgery – as opposed to open surgery – could improve long-term oncological outcomes in patients with advanced, non-metastatic colon cancer (Lacy et al. 2008). However, Lancy and colleagues’ results were based on only 37 and 36 patients in the respective treatment groups (Lacy et al. 2008). This could be a possible reason why their finding was at odds with the results of a comprehensive meta-analysis of RCTs that showed a tendency toward better performance of open surgery in stage II patients (Theophilus et al. 2014). A post-hoc analysis of the European PETACC8 RCT contradicts this comprehensive meta-analysis as laparoscopic surgery was shown to be superior to open surgery in extending 5-year OS in patients with stage III colon cancer (Theophilus et al. 2014; Voron et al. 2021). The authors of the meta-analytic study were aware of their limited power to demonstrate the stage-specific effects of laparoscopic versus open surgery on 5-year OS, and they proposed using big real-world datasets to not only fill this gap but also to ensure the population-level applicability of the RCT results (Theophilus et al. 2014). Such a unifying approach to make decision-making more evidence-based and synergistic is in line with the recommendation of the GRADE working group (Schunemann et al. 2022; Schunemann et al. 2022).

On the other hand, a population-based retrospective cohort study conducted in the United States showed that for patients in stages I and II, laparoscopic colectomy outperformed open colectomy in terms of five-year OS, while there was no difference in stage III patients (Bilimoria et al. 2008). Benz et al. also reported, using nationwide registry data in Germany, that laparoscopic surgery resulted in a higher 5-year OS (Benz et al. 2017). Other observational studies have also shown that the laparoscopic approach provides at least as favorable results as the open approach after five years, even in elderly patients (Horie et al. 2021; Mazaki et al. 2021;  Volkel et al. 2018). It should be noted, however, that it remains likely that immortal time bias and biases arising from other sources can distort estimates presented by observational studies (Bykov et al. 2022; Suissa 2008). As such, the results of observational studies can even be misleading (Emilsson et al. 2018), and caution should be exercised when relying on evidence provided by such study designs. When comparing the two surgical treatment modalities, for example, differences in initial profiles of patients (patient selection) or the exclusion of a significant proportion of patients with incomplete data can lead to biased estimates (Bilimoria et al. 2008; Benz et al. 2017).

Common biases in observational studies such as immortal time bias, can be adequately addressed by emulating a hypothetical target trial (Emilsson et al. 2018; Hernan et al. 2016). Emulating a target trial can help answer comparative effectiveness and safety research questions in the presence of equivocal evidence from RCTs (Hernan and Robins 2016). In contrast to the ideal setting of RCTs, this rigorous study design allows to evaluate the impact of a particular intervention in a pragmatic (real-world) sense (Hernan and Robins 2016). Evidence shows that causal effect estimates derived from a carefully specified and emulated target trial are frequently similar to those obtained from RCTs (Kutcher et al. 2021). A successful emulation of a target trial using observational data can provide reliable evidence, which is particularly relevant when conducting an RCT is not feasible, ethical, or timely or when the generalizability of results from RCTs is unclear (Hernan and Robins 2016). Hernán and colleagues showed that targeted trials can reduce bias risks (immortal time and selection bias) and provide more accurate causal treatment effects (Hernan et al. 2016; Hernan and Robins 2016). This is done by choosing a time-zero that synchronizes follow-up start with eligibility assessment and treatment allocation, and then applying statistical methods to ensure comparability and randomness in treatment allocation (Hernan et al. 2016; Hernan and Robins 2016). Strict implementation of such a procedure at baseline corresponds to randomization in RCTs. Hence target trial emulation provides a novel option to evaluate the long-term comparative effectiveness of therapeutic interventions (Antoine et al. 2023).

In this study, we will use data from the Mecklenburg-Western Pomerania (MV) Cancer Registry to explicitly emulate a hypothetical target trial study that compares 5 year OS between laparoscopic and open surgical treatment strategies in patients diagnosed with stages I–III colon cancer. To this end, a target trial is specified that will randomly assign patients with solitary, non-metastatic colon cancer to either laparoscopic or open surgery, and then emulate this target trial using the cancer registry data over the period of 2008 to 2018. Treatment period- and stage- specific results will also be provided.

## Methods

### Research question

Does laparoscopic colectomy improve the 5-year overall survival rate compared to open colectomy in patients with stage I–III colon cancer?

### Study setting, data source, and study population

A major step toward raising and improving the standard of care in oncological health services in Germany was the nationwide implementation of clinical cancer registries, enacted by the Cancer Early Detection and Registry Act (Krebsfrüherkennungs- und -registergesetz, KFRG) in April 2013 (Federal Ministry of Health of Germany 2013). This emulated target trial study will use data from the Mecklenburg-Western Pomerania Cancer Registry, a registry providing ≥ 90% completeness of coverage (Arndt et al. 2020).

**Fig. 1:**
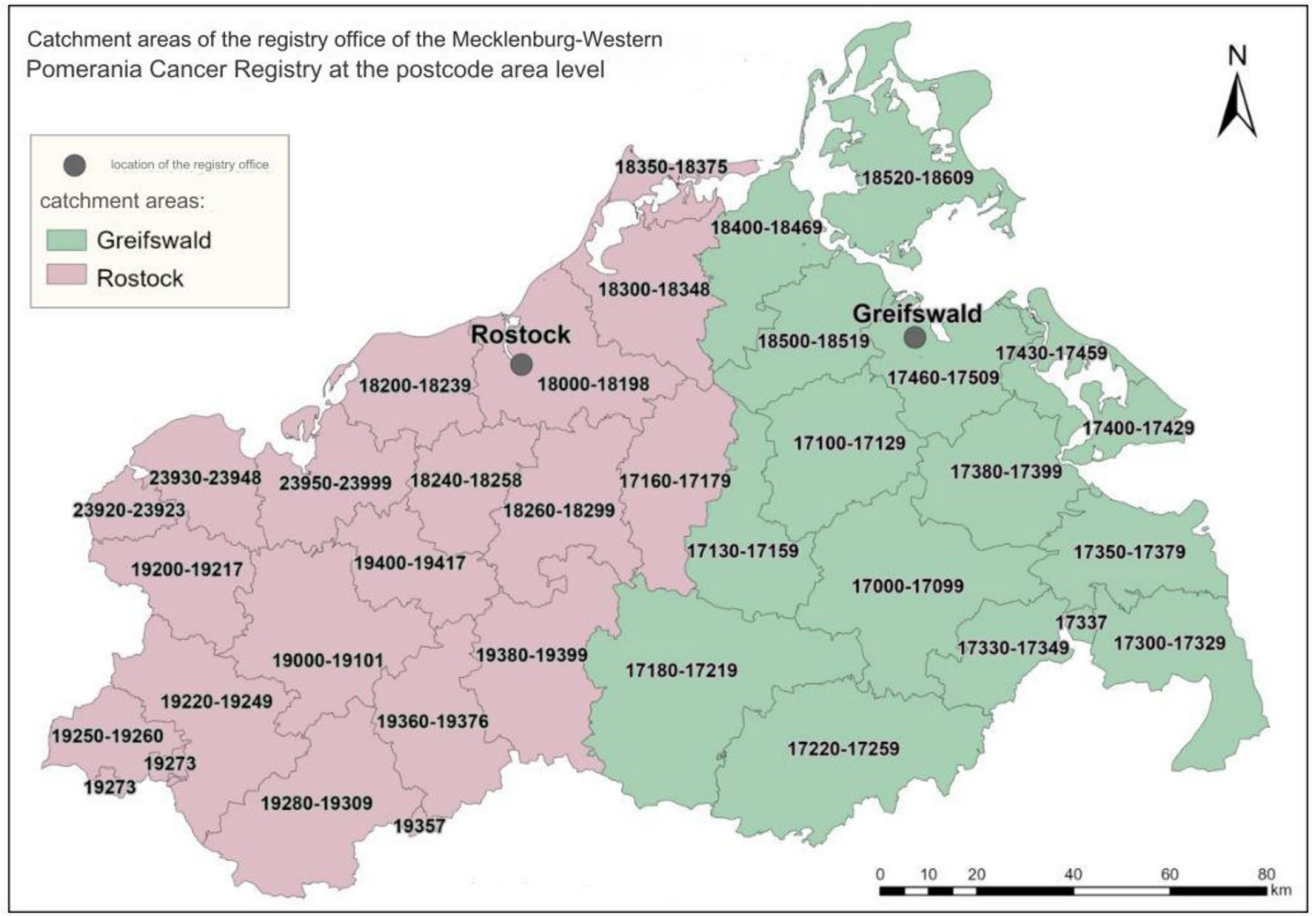
Locations and catchment areas of the two registry offices of the Cancer Registry in Mecklenburg-Western Pomerania by postal code areas, map adapted from the registry’s webpage (The Cancer Registry of Mecklenburg-Western Pomerania 2024)

The estimated population of Mecklenburg-Western Pomerania was 1,629,464 inhabitants as of December 31, 2023 (Statistical Office of Mecklenburg-Western Pomerania 2024). The federal state covers 23,294 square kilometers and is the least densely populated state in Germany, with only 70 people per square kilometer (Statistical Office of Mecklenburg-Western Pomerania 2024). There are currently 38 hospitals in the whole region, just five of which function as certified colorectal cancer treatment centers, Federal Statistical Office of Germany 2024; OncoMap 2024). Compared to all German federal states, Mecklenburg-Western Pomerania had the sixth highest age-standardized incidence rate (ASIR) of colorectal cancer in men and the fourteenth highest in women in the period 2019–2020 (Robert Koch Institute 2024). Between 2008 and 2022, the ASIR of colon cancer in the region was 33.6 per 100,000 population for men and 21.1 per 100,000 population for women 35 (Interactive report of Mecklenburg-Western Pomerania Cancer Registry 2024). Our study population consists of all adult patients with a confirmed diagnosis of primary stages I–III colon cancer between January 1, 2008 and December 31, 2018, who underwent major elective laparoscopic or open surgery (OPS codes 5–455, 5–456, 5–457, and 5–458) during this period (ICD-10-GM 2023). Figure1 below shows the present locations of the Registry’s catchment areas, detailed by the postcode area.

### Design of the target trial and the emulated trial

To answer our research question, this study will follow a two-step implementation process. First, we designed a hypothetical target trial protocol to compare the effect of the surgical treatment modalities on 5 year OS in patients with stages I–III colon cancer. Next, we will use real-world data from the Cancer Registry in Mecklenburg-Western Pomerania to explicitly and validly emulate the prespecified target trial. Table 1 summarizes the components of the specified hypothetical target trial protocol and the corresponding planned emulated trial using population-based cancer registry data. Hernán and Robins outlined seven key components that are essential for specifying target trial emulation studies (Hernan and Robins 2016). It is, however, possible to add other components such as the research question and the aim of the study, as Martinuka and colleagues implemented in their COVID-19 study (Martinuka et al. 2023).

**Table 1:** Components of the hypothetical target trial and the emulated trial

Study component	Target trial	Emulated trial
Research Question	Does laparoscopic colectomy improve the 5 year overall survival rate compared to open colectomy in patients with stage I–III colon cancer?	Same as target trial
Aim	Compare the 5 year OS of patients with stage I–III colon cancer treated by laparoscopic colectomy or open colectomy.	Same as target trial
Design	Phase III, multicenter, open-label, two-parallel-arms RCT	–
Eligibility	Patients with solitary, primary stages I–III colon cancer aged ≥ 18 years at diagnosis, with performance score (Eastern Cooperative Oncology Group, ECOG ≤ 2 or Karnofsky score ≥ 50%), and Charlson comorbidity index (CCI ≤ 2).	Patients with solitary, primary stages I–III colon cancer aged ≥ 18 years at diagnosis, with performance score ECOG ≤ 2 or Karnofsky score ≥ 50%, and laparoscopic or open colectomy within 3 months (90 days) after diagnosis. CCI is not documented by Mecklenburg-Western Pomerania Cancer (MV) Registry center.
Exclusions	Body mass index (BMI) > 35 Kg/m², advanced local disease (T4), adenocarcinomas of the transverse colon and rectum (including that of appendix, hepatic flexure, splenic flexure, overlapping sites and unspecified cancer of the colon), multiple primary colon tumors, prior history of cancer in the past 5 years. However, patients with non-melanoma skin cancer (NMSC) as well as in situ tumors (ICD-10 codes D00-D09) and benign tumors (ICD-10^a^ codes D10-D36) will not be excluded. The following patients will also be excluded: patients who underwent emergency colectomy, patients with gastrointestinal stromal tumor (GIST), neuroendocrine cancers, sarcoma cases, or patients with previous abdominal surgery. Additional excluded cases include robotic colectomies (OPS = 5–987).	Advanced local disease (T4), patients diagnosed with subtypes of colon cancer located on the appendix (C18.1), hepatic flexure (C18.3), transverse colon (C18.4), splenic flexure (C18.5), or overlapping sites of the colon (C18.8), and all unspecified sites (C18.9) – as well as cancer of the rectosigmoid junction (C19) will be excluded. Patients with prior history of cancer in the past 5 years (except NMSC, in situ tumors (ICD-10 codes D00-D09) and benign tumors (ICD-10 codes D10-D36)) will be excluded. In addition, patients with an additional GIST diagnosis, neuroendocrine cancers, sarcoma cases, patients with previous abdominal surgery for malignant indications as well as patients treated with emergency or robotic colectomy (OPS = 5–987) will also be excluded. Since data on BMI, previous surgery for non-malignant indications and CCI are not available in MV cancer registry, we will not use these covariates as exclusion criteria.
Treatment strategies	1. Laparoscopic colectomy 2. Open colectomy	Same as target trial
Grace-period for treatment implementation	–	The first three months after confirmed stages I–III colon cancer diagnosis.
Treatment assignment	Randomization, at baseline	The patient’s assignment to one of the two treatment strategies will be non-random. But randomness will be emulated by cloning patients in both arms.
Outcome	Death from all causes over a follow-up period of 60 months from baseline (start of follow-up)	Same as target trial (5 year OS)
Type of outcome	Time-to-event data	Same as target trial
Follow-up	Follow up begins at diagnosis and the random allocation of patients to either treatment arms and then scheduled follow-up information according to German S3- guideline for colorectal cancer (German Guideline Program in Oncology 2019).	Patients will be followed from time-zero (date of diagnosis) until death from any cause, last date of information or administrative censor date (31.12.2023), whichever will come first.
Censoring	Loss to follow-up, administrative censor date (31.12.2023).	Same as target trial
Causal contrast	Intention-to-treat effect, per-protocol	Observational analogue of the per-protocol effect
Effect measure	Hazard ratio (HR), mean survival time difference	Same as target trial
Analysis plan	Multivariable Cox regression, absolute risk difference and restricted mean survival time (RMST)	Inverse-probability weighted Royston- Parmar parametric survival model. Further to the HR, absolute risk-difference, RMST difference will be calculated.
Covariate adjustments	ϖ Socio-economic characteristics: Age at colon cancer diagnosis (in years) and sex. ϖ Clinical characteristics: UICC^b^ stage (I, II or III), treating institutions, tumor laterality (left or right), treatment period (2008–2009, 2010–2014, 2015–2018), performance score (ECOG = 0, 1, or 2), hospital classification (certified colon center or not), local residual tumor margin status (yes, no, or not assessable), grade (low, intermediate or high), and number of harvested lymph nodes (< 21 or ≥ 21).	ϖ Socio-economic characteristics: same as target trial. ϖ Clinical characteristics: same as target trial.
Sensitivity analysis	Excluding cases with missing values (complete case analysis, CCA)	Sensitivity analyses: - 1) Repeat the same analysis on patients with complete case data - 2) Including all advanced local disease (stage T4N0-2M0) cases - 3) Quantitative bias analysis using the E-value method to evaluate the effect of potential residual confounders (BMI, comorbidity, previous abdominal surgery for non-malignant indications)
Comparator analysis		Comparator analyses: - Comparator 1) DAGs-guided naïve analysis of using fully adjusted cox proportional hazards model - Comparator 2) An Inverse-probability-weighted regression-adjustment (Doubly robust) analysis

^a^ ICD-10 = The International Classification of Diseases, 10th Revision

^b^ UICC = Union for International Cancer Control

The target trial design would include all adult patients (18 years or older) with solitary colon cancer, stages I–III. In addition, patients with a good performance status (Eastern Cooperative Oncology Group, ECOG ≤ 2 or Karnofsky performance status score ≥ 50%), and Charlson comorbidity index (CCI score ≤ 2) will be included. The target trial would exclude patients with an age less than 18 years, a body mass index (BMI) greater than 35 kg/m², prior abdominal surgery for non-malignant indications, treatment after 3 months of diagnosis, cancer of the transverse colon and rectum (within 15 cm of the anal verge on rigid sigmoidoscopy), cancer of the hepatic flexure of the colon, splenic flexure of the colon, overlapping sites of the colon, appendix and unspecified adenocarcinomas of the colon as identified by ICD-10 topographical subcodes, advanced local disease (T4) or adjacent organ invasion, multiple primary colon tumors, prior history of cancer except non-melanoma skin cancer and benign or in situ tumors. Patients with missing ICD-10 topographical subcodes of colon adenocarcinomas, who had an additional diagnosis of gastrointestinal stromal tumor (GIST), neuroendocrine cancers, or sarcomas, and patients who underwent emergency colectomy or robotic colectomy (OPS = 5–987) will also be excluded. In general, our inclusion and exclusion criteria are largely consistent with those of previous RCTs (Bagshaw et al. 2012; Deijen et al. 2017; Lacy et al. 2008). The emulated trial will use almost the same inclusion and exclusion criteria listed in the target trial design, with the exception of BMI, previous history of surgery for non-malignant indications, and CCI, which are not recorded in the cancer registry (see Table1).

The target trial would use either open or laparoscopic surgery as its treatment strategies. The treatments we aspire to evaluate are similar to those of the target trial, but only open and laparoscopic surgeries performed within three months after first colon cancer diagnosis are defined as our treatment strategies. From the perspective of clinical practice, patients are unlikely to undergo surgery on the same day that their colon cancer diagnosis is confirmed. In this context, it is important to determine the time period in which treatment strategies can be implemented to reflect local clinical practice and minimize patient heterogeneity (Hernan and Robins 2017). Since the initiation of surgery after diagnosis requires time, mainly related to diagnostic workup, a time frame (grace-period) must be taken into account in which most colon cancer patients are likely to undergo surgery.

Eligible participants will be randomly assigned to one of the two treatment modalities stratified by tumor stage and hospital and will be unblinded about the treatment they will be receiving in the target trial. In an open-label, multi-institutional, randomized, two-arm phase III trial, Kitano and colleagues randomly assigned patients with stage II–III colon cancer to either laparoscopic or open colectomy one day before surgery (Kitano et al. 2017). Similar to this RCT, our target trial study would randomize the patients one day before the day of surgery, but with a three-months grace-period to allow for successful implementation of the surgical treatment modalities. The incorporation of a grace-period into a pragmatic trial design prevents ill-defined interventions and improves the use of observational data by expanding the pool of patients who can be included in emulated studies while helping capture real-world clinical practice (Hernan et al. 2016; Hernan and Robins 2016). Accordingly, in our emulated study, patients will be classified into either the laparoscopic colectomy group or the open colectomy group for the treatment of colon cancer within the specified grace-period according to the surgical procedures performed as documented in the patients’ cancer registry records. In contrast to the randomized allocation of patients described in the hypothetical RCT mentioned above, the patients in the emulated trial will not be assigned randomly to one of the treatment modalities. Nevertheless, randomness will be emulated by controlling confounders at baseline using inverse-probability weighting methods and/or via cloning of patients in both arms (Hernan and Robins 2016).

Patients will be followed up from the date of assignment to either treatment modality until death from any cause (the outcome of interest), the last date of follow-up, or the administrative censor date (31.12.2023), whichever occurs first. This administrative censor date is chosen to ensure that patients are observed for at least five years. Patient follow-up will be made in accordance with the recommendation of the German S3- guideline for colorectal cancer (German 2019). In the emulated study, we will start following the patients at time-zero, which is defined as the time point at which the eligibility criteria will be met. The initiation of treatment will have to occur within the first three months after the diagnosis of colon cancer (defined grace-period). Follow-up will end on the date of death, last date of follow-up, or 60 months after time-zero, or the administrative censor date Dec. 31, 2023, whichever will occur first. The main outcome will be 5-year OS, which refers to the duration from time-zero until death from any cause. The estimated hazard ratio will be complemented by the mean survival difference (at 1, 3, and 5 years) between the patients treated with laparoscopic and open surgery, respectively. Since our study covers a long period of time (2008 to 2018), treatment period-specific analyses will be performed to mitigate potential bias that may be induced by changes in clinical knowledge and practice over time (Hou et al. 2022).

#### Statistical analysis

Patient characteristics will be summarized using frequencies and percentages for categorical variables, whereas means and standard deviations or medians and interquartile ranges will be used for continuous variables, depending on their distributions.

**Fig. 2:**
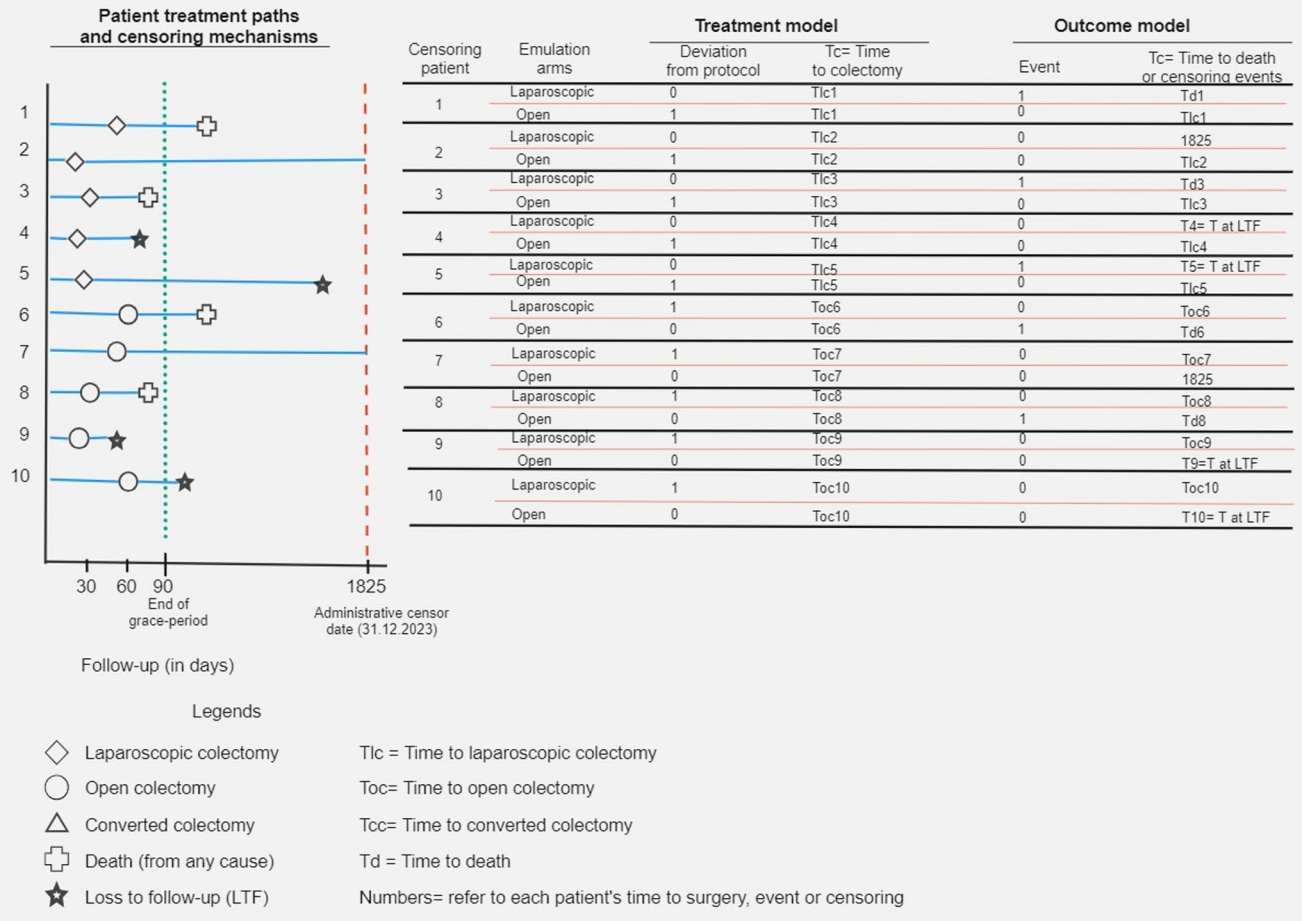
Definition of outcome and survival times for each patient in each emulation arm

We will use the clone-censor-weight design to emulate the hypothetical target trial (Hernan and Robins 2016). Since each patient will be eligible for one of the two surgical treatment modalities during the three-month grace-period, we will first clone (copy) each patient record, and each clone will then be allowed to enter both treatment arms for the duration of the grace-period regardless of the surgical modality they will actually receive later. This will emulate randomization in the specified target trial and addresses the issue of confounding at baseline, as both treatment arms are identical in terms of baseline patient characteristics. Cloning also offers the opportunity for even distribution of survival times (before surgery, for the entire grace-period or any early deaths that may occur before treatment) between the two surgical treatment modalities, allowing us to circumvent the problem of immortal time bias (Hernan and Robins 2016). In the next step, we will censor clones over time if non-compliance with the initially assigned treatment strategy is detected from that timepoint onward. This implies that we will censor clones assigned to laparoscopic colectomy (including converted colectomy) but whose records indicate that they underwent open colectomy within the grace-period, or vice versa. The censoring times will be defined corresponding to the time of each censoring event and will be incorporated into the weighting model described below. Figure2 provides all possible treatment paths and the respective censoring mechanism of the trial emulation for both treatment and outcome models.

The informative censoring (and hence associated selection bias) induced by the implementation of artificial censoring will be adjusted via inverse-probability censoring weighting (IPCW). This will reflect the likelihood of being compliant (uncensored) with the assigned treatment strategy given the confounding factors. However, prior to cloning, an inverse-probability of treatment weighting (IPTW) on the uncloned (original) population will be estimated with the aim of assigning larger weights to patients who will be less likely to undergo laparoscopic colectomy, given the individual constellation of confounding factors. These weights (IPTW and IPCW) will be estimated by running separate models using a logit function and later combined by multiplying both weights. This single combined weight will later allow us to determine an unbiased causal effect of laparoscopic versus open surgical treatment. We will then use absolute standardized differences within ≤ 10% as an indicator of adequate balance of the covariates (confounders) between the two treatment arms. If there will be evidence of imbalance, the weights at the 1st and 99th percentiles will be trimmed to reduce the effect of extreme weights. This will be achieved by replacing all weight values that are below the 1st percentile with the 1st percentile value and by replacing all weight values above the 99th percentile with the 99th percentile value.

Intention-to-treat and per-protocol effects are used as causal contrasts for the target trial design. The intention-to-treat effect refers to the comparative effect of laparoscopic versus open colectomy, independent of subsequent non-compliance that may occur later after time-zero due to conversion from laparoscopic to open colectomy. The per-protocol effect, on the other hand, refers to the comparative effect of exclusive receipt of laparoscopic versus open colectomy. In contrast, the observational-analogue of the intention-to-treat effect will not be estimable in the emulated study because our emulation process will be implementing a grace-period with the cloning-censoring-weighing method. This means that every eligible patient will be assigned to both treatment arms during this period until they will be censored if their treatment modality will deviate from the modality to which they were initially classified. Therefore, our causal contrast of interest in the emulated study will only be the observational-analogue of the per-protocol effect of laparoscopic versus open colectomy performed in the first three months after colon cancer diagnosis.

Finally, we will use an inverse-probability weighted Royston-Parmar parametric survival model (RPpsm) to estimate an unbiased causal effect of laparoscopic versus open surgical treatment on OS via hazard ratio. If incorporating the final weight (derived from IPTW and IPCW) into the RPpsm will not be achieved, we will alternatively use weighted pooled logistic regression to model the outcome. When the event of interest (death from any cause) occurs, that event is considered to be an event solely in the patients’ originally planned treatment arm, in which each patient is initially consistent and remains uncensored at the point in time when the event occurs. On the other hand, the cloned records will be censored at that exact time the event occurs, which is accomplished by censoring indicators. Using weighted RPpsm postestimation tools, OS will be estimated using differences in absolute risk and restricted median survival (RMST) at 1-, 3-, and 5-years and up to 1-, 3-, and 5-years, respectively. To account for the weighting to be applied, point estimates will be accompanied by 95% confidence intervals via non-parametric bootstrapping with 500 replicates. A weighted Kaplan-Meier method will be used to compare time-to-event curves over a 60-months period according to surgical treatment modality. We will compare our results with estimates from the literature. We make use of the CERBOT (Comparative Effectiveness Research Based on Observational Data to Emulate a Target Trial) tool (see Additional file 1) in our emulation process (Zhang et al. 2024).

It is crucial to handle missing data properly to obtain more powerful models resulting in better estimates (Mbona et al. 2023). We will use multiple imputation via the chained equations method to impute missing values in covariates, assuming that observed missing values are missing at random (White and Royston 2009). In the literature, this method is also referred to as “multivariate imputation with sequential regression” and “imputation using fully conditional specifications” (Raghunathan et al. 2001; van Buuren et al. 1999). All variables of the analysis dataset, including the outcome variable, will be used for the imputation model and ten datasets (m = 10) will be imputed.

In the web-based DAGitty environment (https://www.dagitty.net/), we will utilize directed acyclic graphs (DAGs) to determine the minimum necessary adjustment sets of confounders to control for within the comparative multivariable model. This approach mitigates the risk of bias that may arise from conditioning on intermediate covariates or colliders and mediators (Textor et al. 2011; Tonnies et al. 2022). The causal diagram for the comparative multivariable Cox model is complemented by a literature review and discussion to identify confounders, colliders, mediators and prognostic factors (Austin and Stuart 2015; Lederer et al. 2019). By adjusting the minimum sufficient adjustment sets of confounders in our comparative model, we will be able to block any backdoor bias paths (remove relevant confounding) when estimating the total causal effect of surgical treatment modality on all-cause mortality.

We will consider the following baseline characteristics (measured before or at time-zero) as potential confounders: Age at colon cancer diagnosis, sex, year of surgical treatment (classified according to the implementation and update series of the German S3- guideline for colorectal cancer (German Guideline Program in Oncology 2019) as 2008–2009 [pre-implementation], 2010–2014 [first update], or 2015–2018 [second update]), tumor laterality (right-sided [caecum and ascending colon] tumor or left-sided [descending and sigmoid colon] tumor), hospital type (classified as either a registered colorectal cancer center or other group), performance status (ECOG ≤ 2 or Karnofsky performance status score ≥ 50%), and histologic grade (low, intermediate or high), UICC stage (I, II or III), and local tumor size (T-stage). In addition, we will adjust the following two variables that serve as quality indicators of performed surgical procedure: minimum number of harvested lymph nodes (classified as < 21 or ≥ 21) (Choi et al. 2010), and local residual tumor status within 3 months of diagnosis (no (R0), yes (R1 + R2), not assessable or missing). We will also extract the following dates accurate to the month (date of birth, diagnosis, surgery, death, neo/adjuvant therapy initiation, and conversion to open surgery) and other covariates (project specific pseudonym, tumor IDs, cause of death).

#### Sensitivity and comparator analyses

The cancer registry does not collect data on BMI, CCI, or history of previous surgery for non-malignant indications. Given that we will be unable to emulate these potential factors, residual confounding arising from these factors is a major limitation of this study. Thus, we will perform sensitivity analyses.

1. Of residual confounding using the E-value method (VanderWeele and Ding 2017) to provide a quantitative bias analysis for potential residual confounders (BMI, comorbidity, and previous abdominal surgery for non-malignant indications), and.
2. After excluding patients with missing data to further evaluate the potential for selection bias that would otherwise have occurred if such cases were excluded. Further sensitivity analysis will be performed by including all patients with advanced local disease (stage T4N0-2M0).

In addition, a comparative analysis of the same but unemulated study population that started treatment at any point in time will be performed via classical Cox regression to assess the influence of our emulation process. As identified by the DAGs, the treatment effect, which adjusts for “age, grade, performance status, and tumor laterality” in this comparative model will be unbiased. Through this, we will practically examine whether the conclusion of the comparative analysis actually differs from that of the emulated study (see Additional file 2). Another comparative analysis of the same emulated population will be performed via the inverse-probability weighted regression adjustment (doubly robust) method. This approach combines the outcome modeling strategy of regression adjustment and the treatment modeling strategy of inverse-probability weighting (Smith et al. 2022). It is referred to as “doubly robust” because although it requires running two models, of which only one—the treatment or outcome model—needs to be correctly specified to obtain an unbiased estimate of the treatment effect (Smith et al. 2022).

All our analyses will be performed in STATA (version 18.0, College Station, TX: StataCorp LLC), and statistically significant associations will be declared at a p-value of less than 0.05.

## Discussion

This study aims to causally evaluate the effect of laparoscopic versus open colectomy on 5-year OS by applying a target trial emulation methodology with a clone-censor-weight approach. Our study will be based on data from the Mecklenburg-Western Pomerania Cancer Registry, focusing on patients diagnosed with stage I – III colon cancer between 2008 and 2018. Disease stage- and treatment-period specific results will also be provided. A limitation of this study is potential residual confounding due to missing BMI, comorbidity and previous abdominal surgery data in the registry. To address this, a quantitative bias analysis using the E-method will be conducted. The analysis will increase robustness by reducing biases commonly found in observational studies (Emilsson et al. 2018; Hernan et al. 2016). This enriches the existing evidence from population-based data and contributes to a better understanding of the causal impact of surgical modalities on the long-term survival of patients with colon cancer. The application of such novel causal analysis approaches may further increase the utility of big administrative databases for answering pressing clinical questions (Hernan and Robins 2016).

## Conclusions

The results of this study will substantiate existing evidence on the comparative effectiveness of laparoscopic versus open colectomy in patients with stage I–III colon cancer. This may guide clinical decisions as to whether a laparoscopic approach is as safe as an open approach in terms of improving 5 year overall survival in these patient groups.

## Supplementary Information

Below is the link to the electronic supplementary material.

Supplementary material 1 (DOCX 636.4 kb)

Supplementary material 2 (DOCX 31 kb)

## Data Availability

No datasets were generated or analysed during the current study.

## Abbreviations

ASIR: Age-standardized Incidence Rate
BMI: Body Mass Index
CCI: Charlson Comorbidity Index
CERBOT: Comparative Effectiveness Research Based on Observational Data to Emulate a Target Trial
DAGS: Directed Acyclic Graphs
ECOG: Eastern Cooperative Oncology Group
GRADE: Grading of Recommendations Assessment, Development and Evaluation
GIST: Gastrointestinal Stromal Tumor (GIST)
ICD-10: The International Classification of Diseases, 10th Revision
KFRG: Cancer Early Detection and Registry Act (Krebsfrüherkennungs- und -registergesetz)
MV: Mecklenburg-Western Pomerania (Mecklenburg-Vorpommern)
OPS: Operation and procedure (Operationen- und Prozedurenschlüssel)
OS: Overall Survival
RCTs: Randomized Controlled Trials
RPpsm: Royston‒Parmar parametric survival model
RMST: Restricted Mean Survival Time
UICC: Union for International Cancer Control
