# Supplementary material for: Comparative effectiveness of laparoscopic versus open colectomy in colon cancer patients: a study protocol for emulating a target trial using cancer registry data
Source: J Cancer Res Clin Oncol. 2025 Jan 11;151(1):34. doi: 10.1007/s00432-024-06057-x (PMC11724780; doi:10.1007/s00432-024-06057-x)
Supplement: Supplementary file 1 — Supplementary material 1 (DOCX 636.4 kb) [file 432_2024_6057_MOESM1_ESM.docx]

Table 1. Evidence-base for the DAG model

| Covariates | Age | Sex | BMI | Comorbidity | Hospital type | Surgeon’s skill | Tumor laterality | Resection laterality | Grade | Stage | Harvested LNs | Genes | Emergency operation | Surgical type | Mortality  (all-cause) |
| --- | --- | --- | --- | --- | --- | --- | --- | --- | --- | --- | --- | --- | --- | --- | --- |
| Age | - |  | [1] |  |  |  | [2, 3] |  |  |  |  |  |  |  |  |
| Sex |  | - |  | [4, 5] |  |  |  |  |  |  |  |  |  | [6] | [7] |
| BMI |  |  | - |  |  |  |  |  |  |  |  |  |  |  | [8] |
| Comorbidity |  |  |  | - |  |  |  |  |  | [9] |  |  |  |  | [10] |
| Performance status |  |  |  | x |  |  |  |  |  |  |  |  |  |  | [11] |
| Hospital type |  |  |  |  | - |  |  |  |  |  |  |  |  |  |  |
| Surgeon’s skill |  |  |  |  |  | - |  |  |  |  |  |  |  | [12] | [13] |
| Tumor laterality |  |  |  | [2, 14] |  |  | - |  |  | [2, 3] | [15, 16] |  |  |  | [3, 15] |
| Resection laterality |  |  |  |  |  |  |  | - |  |  |  |  |  | [17] | [18] |
| Grade |  |  |  |  |  |  |  |  | - |  |  |  |  |  |  |
| Stage |  |  |  |  |  |  |  |  |  | - |  |  |  |  |  |
| Harvested LNs |  |  |  |  |  |  |  |  |  |  | - |  |  |  | [15, 16] |
| Genes |  |  |  | [19] |  |  | [20, 21] |  |  | [21] |  | - |  |  | [21, 22] |
| Emergency operation |  |  |  |  |  |  |  |  |  |  |  |  | - |  | [23] |
| Surgical type |  |  |  |  |  |  |  |  |  | [6] |  | [21] |  | - | The research question |

x= hypothesized link

# References

1. van Eeghen, E.E., et al., *Impact of age and comorbidity on survival in colorectal cancer.* J Gastrointest Oncol, 2015. **6**(6): p. 605-12.

2. Kwaan, M.R., et al., *Are right-sided colectomy outcomes different from left-sided colectomy outcomes?: study of patients with colon cancer in the ACS NSQIP database.* JAMA Surg, 2013. **148**(6): p. 504-10.

3. Meguid, R.A., et al., *Is there a difference in survival between right- versus left-sided colon cancers?* Ann Surg Oncol, 2008. **15**(9): p. 2388-94.

4. van Leersum, N.J., et al., *Increasing prevalence of comorbidity in patients with colorectal cancer in the South of the Netherlands 1995-2010.* Int J Cancer, 2013. **132**(9): p. 2157-63.

5. Qiu, H., et al., *Comorbidity Patterns in Patients Newly Diagnosed With Colorectal Cancer: Network-Based Study.* JMIR Public Health Surveill, 2023. **9**: p. e41999.

6. Benz, S., et al., *Laparoscopic surgery in patients with colon cancer: a population-based analysis.* Surg Endosc, 2017. **31**(6): p. 2586-2595.

7. Losurdo, P., et al., *Survival and long-term surgical outcomes after colorectal surgery: are there any gender-related differences?* Updates Surg, 2022. **74**(4): p. 1337-1343.

8. Renfro, L.A., et al., *Body Mass Index Is Prognostic in Metastatic Colorectal Cancer: Pooled Analysis of Patients From First-Line Clinical Trials in the ARCAD Database.* J Clin Oncol, 2016. **34**(2): p. 144-50.

9. Zafar, S.Y., et al., *Comorbidity, age, race and stage at diagnosis in colorectal cancer: a retrospective, parallel analysis of two health systems.* BMC Cancer, 2008. **8**: p. 345.

10. Michalopoulou, E., et al., *Impact of comorbidities at diagnosis on the 10-year colorectal cancer net survival: A population-based study.* Cancer Epidemiol, 2021. **73**: p. 101962.

11. Strandberg Holka, P., et al., *Significance of poor performance status after resection of colorectal liver metastases.* World J Surg Oncol, 2018. **16**(1): p. 3.

12. Miskovic, D., et al., *Learning curve and case selection in laparoscopic colorectal surgery: systematic review and international multicenter analysis of 4852 cases.* Dis Colon Rectum, 2012. **55**(12): p. 1300-10.

13. Brajcich, B.C., et al., *Association Between Surgical Technical Skill and Long-term Survival for Colon Cancer.* JAMA Oncol, 2021. **7**(1): p. 127-129.

14. Groene, S.A., et al., *Right Versus Left-Sided Colectomies: A Comparison of Outcomes.* Am Surg, 2016. **82**(7): p. 580-7.

15. Lee, L., et al., *Lower survival after right-sided versus left-sided colon cancers: Is an extended lymphadenectomy the answer?* Surg Oncol, 2018. **27**(3): p. 449-455.

16. Yang, L., et al., *Prognostic value of total number of lymph nodes retrieved differs between left-sided colon cancer and right-sided colon cancer in stage III patients with colon cancer.* BMC Cancer, 2018. **18**(1): p. 558.

17. Nfonsam, V., et al., *Analyzing clinical outcomes in laparoscopic right vs. left colectomy in colon cancer patients using the NSQIP database.* Cancer Treat Commun, 2016. **8**: p. 1-4.

18. Bourakkadi Idrissi, M., et al., *Left-Sided Colon Cancer and Right-Sided Colon Cancer: Are They the Same Cancer or Two Different Entities?* Cureus, 2023. **15**(4): p. e37563.

19. Ljubic B, et al., *Comorbidity network analysis and genetics of colorectal cancer.* Informatics in Medicine Unlocked, 2020. **21**.

20. Benedix, F., et al., *Comparison of 17,641 patients with right- and left-sided colon cancer: differences in epidemiology, perioperative course, histology, and survival.* Dis Colon Rectum, 2010. **53**(1): p. 57-64.

21. Meng, M., et al., *The current understanding on the impact of KRAS on colorectal cancer.* Biomed Pharmacother, 2021. **140**: p. 111717.

22. Kamphues, C., et al., *The interplay of KRAS mutational status with tumor laterality in non-metastatic colorectal cancer: An international, multi-institutional study in patients with known KRAS, BRAF, and MSI status.* J Surg Oncol, 2021. **123**(4): p. 1005-1014.

23. Stormark, K., et al., *Nationwide implementation of laparoscopic surgery for colon cancer: short-term outcomes and long-term survival in a population-based cohort.* Surg Endosc, 2016. **30**(11): p. 4853-4864.


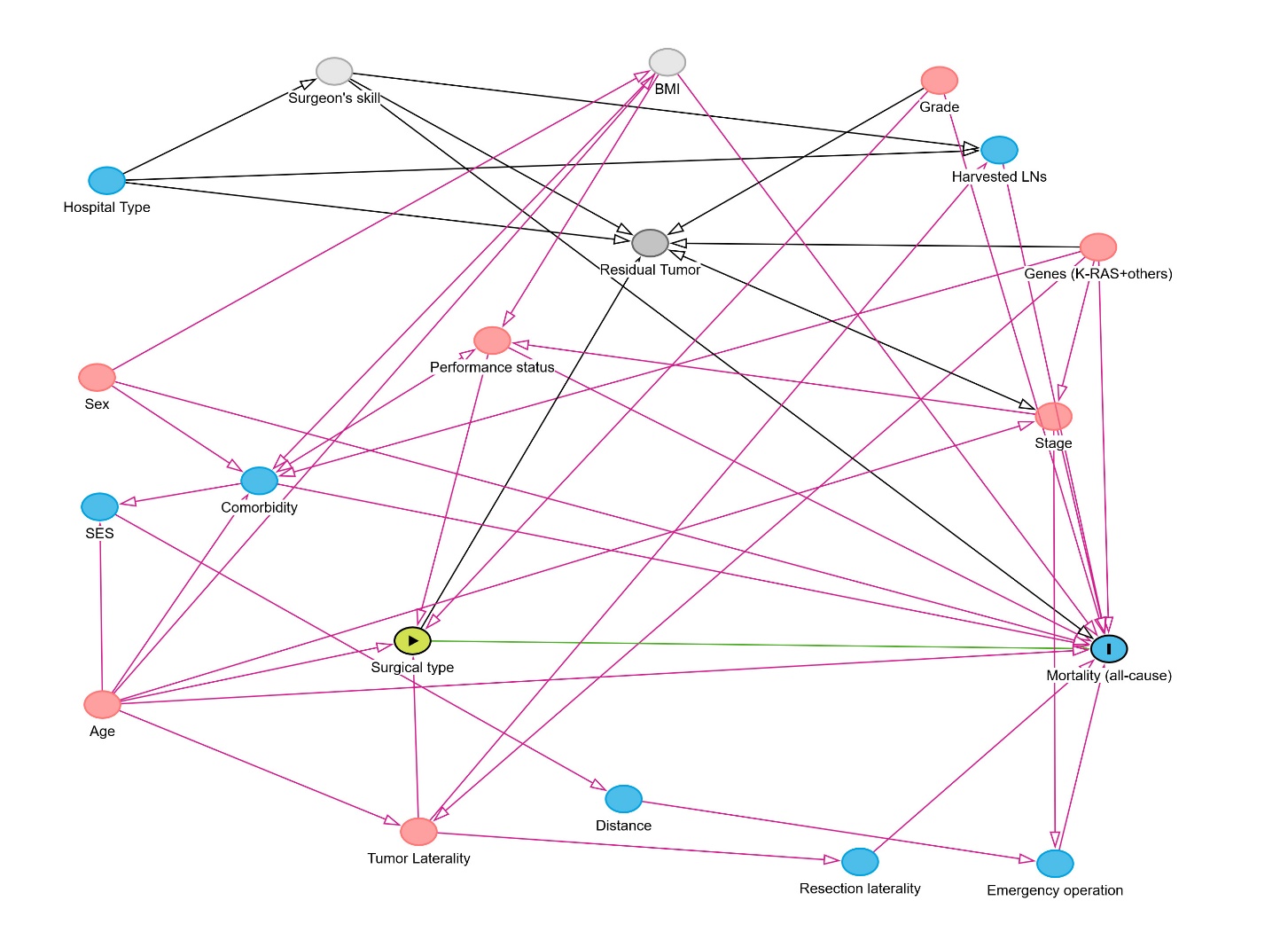


Fig. 1 Direct acyclic graph (DAG) displaying all bias paths in the covariate system for estimating the total effect of surgical treatment modality on all-cause mortality in patients with non-metastatic colorectal cancer in MV, Germany

*Legend: Pink color shows all the confounders and biasing paths that has to be controlled; light gray circles show unmeasured (unobserved) covariates; blue circles show ancestors of the outcome with no link to the primary exposure 8surgical treatment modality) and these factors do not need to be adjusted: green lien shows the total causal effect of the primary exposure (surgical treatment modality) on all-cause mortality outcome*

In the DAG above, the model identified a minimally sufficient adjustment set of confounders required for conditioning in our analysis. The confounders listed in the adjustment set includes “**Age, Grade, Performance status, Tumor Laterality**”. Adjusting these confounders will block all the biasing back-door paths in estimating the total effect of surgical treatment type (open or laparoscopic colectomy) on all-cause mortality. Thereby, we can see from the DAG model below (Fig. 2) that a model which conditions on “**Age, Grade, Performance status, Tumor Laterality**” in the estimation of the total effect of surgical treatment modality on all-cause mortality is unbiased, meaning there is no biasing path (which initially were pink colored) once we adjust these relevant confounders (the white circles in Figure 2 below for these confounding covariates indicate that these relevant confounders are fully adjusted). Although we do not have data on the unmeasured covariate “surgeon’s skill”, this also does not induce bias either because we can account for the effect of the ancestor covariate “hospital type”. The same applies to the unmeasured covariate BMI, since we have data and are able to adjust the effect of its ancestral covariate, i.e., age of the patients.


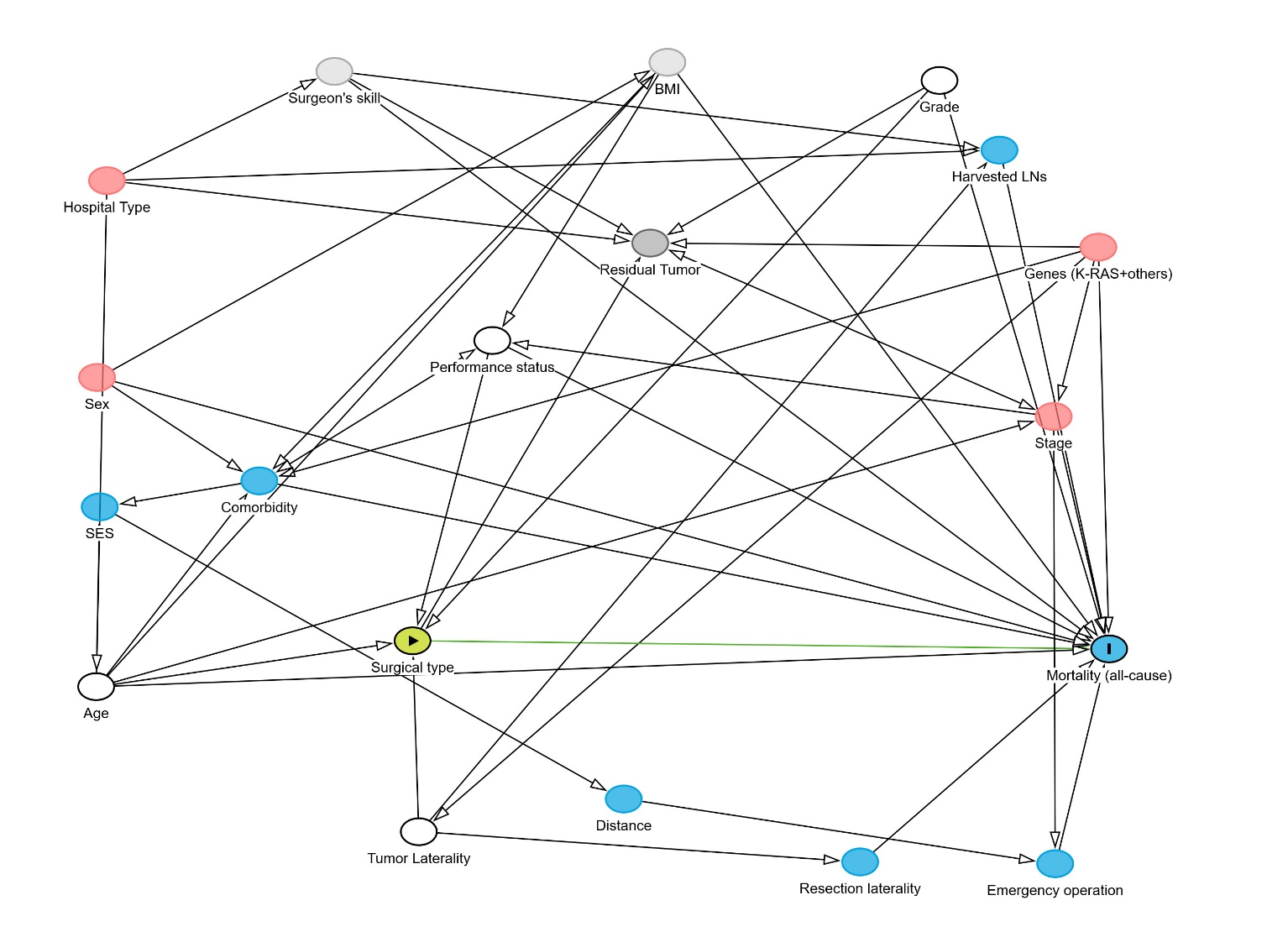


Fig. 2 Direct Acyclic Diagram (DAG) after correct adjustment of the confounders (Age, Grade, Performance status, Tumor Laterality) in the estimation of total effect of surgical treatment modality on all-cause mortality among non-metastatic colon cancer patients in MV, Germany
