# Supplementary material for: Comparative effectiveness of laparoscopic versus open colectomy in colon cancer patients: a study protocol for emulating a target trial using cancer registry data
Source: J Cancer Res Clin Oncol. 2025 Jan 11;151(1):34. doi: 10.1007/s00432-024-06057-x (PMC11724780; doi:10.1007/s00432-024-06057-x)
Supplement: Supplementary file 2 — Supplementary material 2 (DOCX 31 kb) [file 432_2024_6057_MOESM2_ESM.docx]

**What is the comparative effectiveness of laparoscopic versus open surgery in non-metastatic colon patients in terms of 5-year overall survival?**

The study will use the following data sources: Mecklenburg-Western Pomerania Cancer Registry for the calendar period January 1, 2008 to December 31, 2018 and Hospital Classification Data from the Oncomap database

# Module 1 Report: Eligibility criteria

Table 1. Eligibility Criteria of Study Population

| Eligibility criteria | Response | File Name | Variable Name | Variable Values | Concerns |
| --- | --- | --- | --- | --- | --- |
| 1. Who is your target population? | Patients with solitary, non-metastatic colon cancer (adenocarcinoma of the cecum, ascending colon, descending colon, or sigmoid colon) |  |  |  |  |
| 2. During what time period will you identify study subjects? | From1 Jan 2008 and 31 December 2018 | Mecklenburg-Vorpommern Clinical Cancer Registry |  |  |  |
| 3.1. Age | 18 years or older | “ |  |  | Age needs to be calculated as age at diagnosis |
| 5.1. What medical condition(s) do you want to include? | Diagnosis of colon cancer and treatment by either laparoscopic or open colectomy | Mecklenburg-Vorpommern Clinical Cancer Registry |  |  |  |
| 5.2. What medical condition(s) do you want to exclude? | Advanced local disease (T4) or adjacent organ invasion | “ |  |  |  |
|  | Multiple primary colon tumors | “ |  |  |  |
|  | Prior history of cancer in the past 5 years except non-melanoma skin cancer (NMSC) as well as in situ tumors (ICD-10 codes D00-D09) and benign tumors (ICD-10 codes D10-D36) | “ |  |  |  |
|  | An additional diagnosis of gastrointestinal stromal tumor (GIST), neuroendocrine cancers, or sarcomas, and patients who underwent emergency colectomy or robotic colectomy (OPS= 5-987) | “ |  |  |  |
| 8. Any requirements for provider type or site of care? | Patients who do not live in the state of Mecklenburg-Western Pomerania at the time of diagnosis will also be excluded | “ |  |  |  |
| 9. Additional eligibility criteria not listed above? | body mass index (BMI) greater than 35 Kg/m² |  |  |  | No BMI data in the database |
|  | Patients with missing ICD-10 topographical subcodes of colon adenocarcinomas  will also be excluded. | Mecklenburg-Vorpommern Clinical Cancer Registry | icd10n | C18.0 to C18.9 |  |

# Module 2 Report: Outcomes

Table 2. Study outcomes to be evaluated in the study

| Outcomes | Response | File Name | Variable Name | Variable Values | Concerns |
| --- | --- | --- | --- | --- | --- |
| 1. What type is it? | failure-time |  |  |  |  |
| 2. Define your outcome | Time to death from all causes (overall survival) | Mecklenburg-Vorpommern Clinical Cancer Registry | Todesursachen and Sterbedatum | According to ICD-10 code and related date of death (DD.MM.YYYY) |  |

# Module 3 report: Follow-up Period

Each eligible study subject either experiences the outcome or is censored. Study subjects are followed from baseline to when study outcome (Table 2) or one of the censoring events (Table 3) occurs, whichever happens earlier.

Table 3. Censoring events

| Follow-up Period | Response | File Name | Variable Name | Variable Values | Concerns |
| --- | --- | --- | --- | --- | --- |
| 1.1 Administrative end of follow-up | 31 December 2018 | NA | NA | 60 months after baseline, and variable name defined when defining end of follow-up |  |
| 1.2 Loss to follow-up | Last date of follow-up | Mecklenburg-Vorpommern Clinical Cancer Registry |  |  |  |
| 1.3 Other censoring events | Treatment strategy noncompliance during the 90 days of window period |  |  |  |  |
| 1.4 Do you expect that censoring may result in selection bias? | yes |  |  |  |  |

# Module 4 Report: Treatment Strategies

The study compares two treatment strategies: Laparoscopic surgery vs. Open surgery. In the target trial, each participant will be randomized to either of the two treatment strategies. In the observational study, eligible patients are classified into 2 groups in which data are consistent with those defined in the target trial. Table 3 below summarizes for each treatment strategy the target trial protocol as well as how this is defined using observational data.

Table 4. Treatment strategies to be compared in the study

| Treatment Strategies | Response | File Name | Variable Name | Variable Values | Concerns |
| --- | --- | --- | --- | --- | --- |
| 1. Strategy label | Laparoscopic surgery |  |  |  | In practice, patients do not undergo surgery on the day they are diagnosed because of diagnostic and administrative reasons. |
| 2.1. Does this treatment strategy consist of an intervention that happens at baseline only? | point |  |  |  |  |
| Describe the intervention in such a way that other people can implement it | Treatment by laparoscopic surgery | Mecklenburg-Vorpommern Clinical Cancer Registry | OPS | OPS code values for laparoscopic colectomy |  |
| Do you allow a certain period of time (grace period) to implement this intervention? | yes |  |  |  |  |
| Do you allow a certain period of time (grace period) to implement this intervention? | Surgery performed within 90 days after colon cancer diagnosis | “ | Tumor Diagnosedatum, icd10n, OPS, OPS datum | laparoscopic colectomy within 90 days after diagnosis |  |
| 1. Strategy label | Open surgery |  |  |  | In practice, patients do not undergo surgery on the day they are diagnosed because of diagnostic and administrative reasons. |
| 2.1. Does this treatment strategy consist of an intervention that happens at baseline only? | point |  |  |  |  |
| Describe the intervention in such a way that other people can implement it | Treatment by open surgery | Mecklenburg-Vorpommern Clinical Cancer Registry | OPS | OPS code values for open colectomy |  |
| Do you allow a certain period of time (grace period) to implement this intervention? | yes |  |  |  |  |
| Do you allow a certain period of time (grace period) to implement this intervention? | Surgery performed within 90 days after colon cancer diagnosis |  | Tumor Diagnosedatum, icd10n, OPS, OPS datum | open colectomy within 90 days after diagnosis |  |

# Module 5: Adjustment Variables

Although in Modules 1-4 you have attempted to emulate a target trial with regard to eligibility criteria, treatment strategies, follow-up and outcomes, substantial differences remain between your observational study and target. The most prominent difference is that treatment was not assigned at random in your observational ‘trials’. Rather, those who were assigned to Strategy 1 were different from those who were assigned to Strategy 2, i.e., there is confounding by indication. These differences between the two study groups are not expected in a randomized trial, and thus no confounding adjustment is usually performed in randomized trials. In contrast, estimating causal effects from your ‘trials’ requires confounding adjustment. You selected the variables in Table 5 as potential confounders based on a priori knowledge and a review of observational studies on the effectiveness of treatment strategies. To emulate the random assignment of the treatment strategies at baseline, you need to adjust for all confounding factors required to ensure comparability (exchangeability) of the groups defined by initiation of the treatment strategies. Table 5 below lists baseline confounding factors adjusted in the study.

Table 5. Adjustment variables

| Adjustment Variables | Response | File Name | Variable Name | Variable Values | Concerns |
| --- | --- | --- | --- | --- | --- |
| 2.1. Age | Age at diagnosis, in years | Mecklenburg-Vorpommern Clinical Cancer Registry | calculated from Tumor Diagnosedatum and Geburtsdatum | Diagnosedatum minus Geburtsdatum, calculated in years |  |
| 2.2. Sex | Sex | “ | Geschlecht | M for male, F for female |  |
| 3. Baseline variables, including quality indicators of performed surgical procedures, related to patient outcomes |  |  |  |  |  |
|  | Surgical treatment period | “ | Computed from OP_DATUM1 | classified according to the implementation and update series of the German S3- guideline for colorectal cancer: 2008-2009 [pre-implementation], 2010-2014 [first update], or 2015-2018 [second update] |  |
|  | Performance status | “ | ECOG1 | 0, I, II (ECOG≤2 or Karnofsky performance status score ≥50%) |  |
|  | Hospital classification | Oncomap database, accessible at https://www.oncomap.de/ | hosptype | classified as either a registered colorectal cancer center or other group |  |
|  | Local residual tumor margin status within 90 days after diagnosis | Mecklenburg-Vorpommern Clinical Cancer Registry | lokalen Residualstatus | no (R0), yes (R1+R2), not assessable or missing |  |
|  | Stage | “ | uicc | I, II, III |  |
|  | Grade | “ | GRADING | L = low grade (G1 or G2) M = intermediate (G2) H = high grade (G3 or G4) |  |
|  | Local tumor size | “ | T_gruppe | T-stage |  |
|  | Minimum number of harvested lymph nodes | “ | LK_UNTERSUCHT1 | classified as < 21 or ≥ 21 |  |
|  | Tumor laterality | “ | Tumor Seitenlokalisation | right-sided [caecum and ascending colon] tumor or left-sided [descending and sigmoid colon] tumor |  |
| 4. Additional Baseline Variables |  |  |  |  | Not measured |
| 7. Do you have an instrument variable? If yes, please specify | no |  |  |  |  |
| 9. Which variables are potentially important, but are not measured in your data? | Body mass index |  |  |  | Not measured |
|  | Charlson Comorbidity Index |  |  |  | Not measured |
|  | Abdominal surgery for non-malignant indications |  |  |  | Not measured |

# Statistical analysis plan

**Based on your input from previous modules, we have recommended the following analytic approaches applicable to your study:**

Because treatment strategies to be compared are point interventions, both standard methods (e.g., regression analysis, stratification, matching, propensity score, etc.) and advanced analytical methods (e.g., g-methods and doubly robust methods) can be considered. Please go to the Resources page to view g-methods tutorials regarding conceptual basis, assumptions, and implementation tools. Inverse-probability weighting methods is also recommended to adjust for informative loss-to-follow up.

Analytical techniques to handle grace period should also be considered. During the grace period specified in your target trial, an individual's observational data is consistent with more than 1 treatment strategy (see case study 1). One way is to randomly assign the individual to one of them. Another way is to create exact copies (clones) with each copy assigned with a different strategy. Each copy is censored when it deviates from its originally assigned treatment strategy, often referred to as artificial censoring. An intention-to-treat analysis is not suitable because each individual may have been assigned to several strategies at baseline and thus have several copies. Additionally, the potential selection bias introduced by artificial censoring needs to be corrected by appropriate adjustment for time-varying factors (e.g., via inverse probability weighting).

Because your study outcome is a time-to-failure variable, survival analysis should be considered. Survival curves should also be constructed. There are always alternative methods to address the same questions. Sensitivity analyses are always required due to pros and cons associated with each method.
